# Prospects and fundamental limits in exceptional point-based sensing

**DOI:** 10.1038/s41467-020-16373-8

**Published:** 2020-05-15

**Authors:** Jan Wiersig

**Affiliations:** Institut für Physik, Otto-von-Guericke-Universität Magdeburg, Postfach 4120, 39016 Magdeburg, Germany

**Keywords:** Optical sensors, Microresonators, Imaging and sensing, Nonlinear optics

## Abstract

Exotic degeneracies in open quantum systems, so-called exceptional points, show rich physics and promise new applications, such as sensors with greatly enhanced response. Recent research on laser gyroscopes has uncovered limits of such sensors due to excess quantum noise.

## Exceptional points

In quantum mechanics, physical observables are represented by Hermitian operators, e.g. the energy is represented by the Hamiltonian. The Hermiticity of the operator ensures that its eigenvalues, i.e. the possible outcomes of measurements, are real. Notwithstanding this, non-Hermitian Hamiltonians can describe the time evolution of open and nonconservative quantum (and classical wave) systems. The eigenvalues of a non-Hermitian Hamiltonian are in general complex numbers, but the imaginary part has a simple physical interpretation as a decay constant, which is related to the finite width of a spectral line. The eigenstates of the Hamiltonian, that is, the energy eigenstates, are quasi-stationary wave functions. This concept has been introduced by G. Gamow almost a century ago to describe the radioactive decay.

**Fig. 1:**
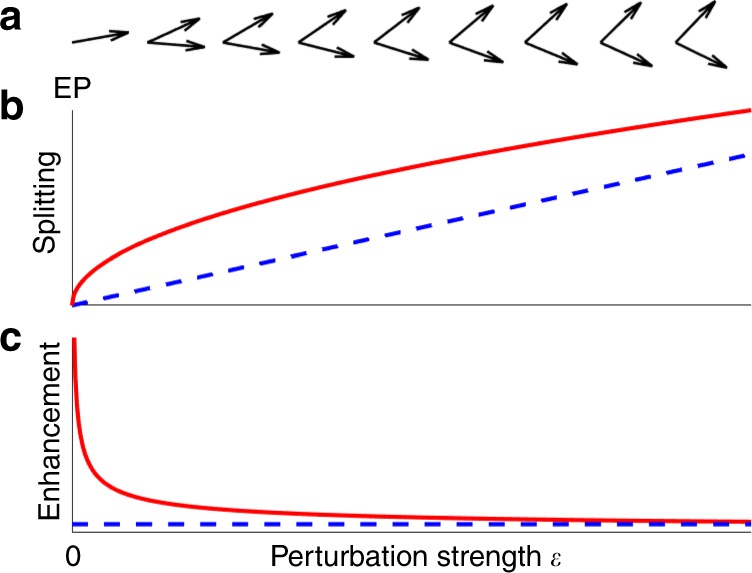
Nonorthogonality of energy eigenstates and enhanced energy splitting near an exceptional point. **a** Illustration of nonorthogonal energy eigenstates as eigenvectors in Hilbert space. At a second-order exceptional point (EP), exactly two eigenvectors become parallel; thus, the number of linearly independent eigenvectors is reduced by one. **b** Energy splitting vs. perturbation strength *ε* for the EP (red solid curve) and a conventional degeneracy (blue dashed curve). **c** Splitting enhancement factor of an EP-based sensor (red solid curve) vs. *ε*. The blue dashed line marks the reference value of unity.

The renewed strong interest in non-Hermitian Hamiltonians is due to further, more intriguing consequences of the non-Hermiticity. For example, the energy eigenstates are not orthogonal to each other. At certain points in parameter space, so-called exceptional points^1^, two or more eigenstates, and their associated eigenvalues, even coalesce, i.e. the corresponding vectors in Hilbert space become parallel as illustrated in Fig. 1a. This peculiar behavior does not happen at conventional degeneracies known from conservative systems where only the eigenvalues degenerate but the eigenstates can always be chosen to be orthogonal. The physical existence of exceptional-point degeneracies and potential applications have been demonstrated experimentally on various platforms, most notably in optics and photonics^2^.

## Exceptional point-based sensing

One remarkable and counterintuitive feature of exceptional points is the strong response of the degenerate eigenvalues to perturbations^1^. When a second-order exceptional point at which exactly two eigenstates coalesce is subjected to a weak perturbation of strength *ε*, then the resulting eigenvalue splitting is proportional to the square root of *ε*, which is larger than the linear splitting for conventional degeneracies as depicted in Fig. 1b. The splitting enhancement factor defined as the quotient of the enhanced splitting and the reference splitting at a corresponding conventional degeneracy is inversely proportional to the square root of *ε* and therefore diverges as *ε* goes to zero (Fig. 1c). This drastically enhanced splitting can be exploited for sensing^3^ which has been demonstrated in a range of proof-of-principle experiments. The first experiments have been on optical microcavities used for light confinement^4,5^, where the record splitting enhancement factor reached an impressive value of 23.

## Limits set by quantum noise

The exceptional point-based sensors fabricated previously use waves (e.g. electromagnetic or acoustic waves) in the classical domain. The impact of quantum noise on exceptional point-based sensors has been controversially debated in recent theoretical studies (see e.g. refs. ^6,7^). The first experiment in this direction has now been done by Wang et al.^8^ extending their preceding work on Brillouin laser gyroscopes^9^ by a detailed study of the noise near the exceptional point. Laser gyroscopes are being used nowadays in commercial aircraft or in carrier rockets for navigation. The principle of operation is the Sagnac effect in a rotating ring-type cavity, which is the opposite shift of the frequencies of counterpropagating optical waves in the cavity. It scales linearly with the rotational velocity Ω.

**Fig. 2:**
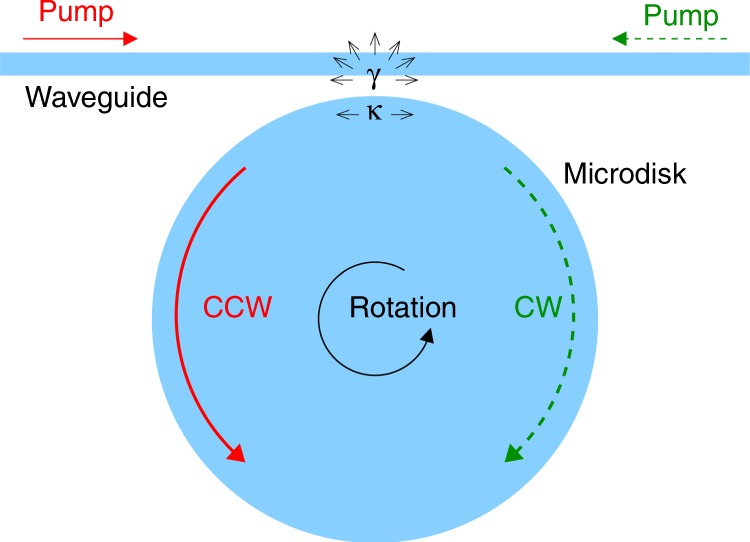
Simplified schematic of the Brillouin laser gyroscope in the experiment by Wang et al.^8^. Laser action through the Brillouin process is achieved in an optically pumped microdisk. As required by the Brillouin phase matching condition, the excited laser waves propagate in a direction opposite to the corresponding pump waves. The evanescent coupling to the waveguide causes scattering losses (rate *γ*) and backscattering (rate *κ*) of clockwise (CW) and counterclockwise (CCW) propagating waves. The waves experience opposite Sagnac frequency shifts used to sense a rotation of the system.

The cavity under study is a silica microdisk (see Fig. 2), which exhibits long-lived cavity modes with resonant frequencies—the optical analog of energy eigenstates and values—appearing in pairs of clockwise and counterclockwise propagating waves. Their frequencies *ω*_1_ and *ω*_2_ are normally equal, but here slightly detuned because of stimulated Brillouin processes^9^ which, moreover, lead to lasing action resulting in ultranarrow spectral lines highly suitable for precise measurements of small frequency shifts. The coupling of the cavity to a waveguide used to pump the Brillouin laser modes induces scattering losses with the rate *γ* and (dissipative) backscattering of the counterpropagating waves with the rate *κ*. All of these properties are described by the non-Hermitian Hamiltonian

1 $$\hat{H} = \left( {\begin{array}{*{20}{c}} {\omega }_{1}-i\gamma & i\kappa \\ i\kappa & {\omega }_{2}-i\gamma \end{array}} \right),$$

which exhibits an exceptional point at ∣*ω*_2_ − *ω*_1_∣ = 2*κ* > 0. A rotation of the gyroscope with velocity Ω changes the frequencies *ω*_1_ and *ω*_2_ differently. Hence, the Hamiltonian at the exceptional point is perturbed with strength proportional to Ω resulting in an enhanced frequency splitting proportional to the square root of Ω. The experiment of Wang et al.^8^ has clearly demonstrated this enhanced response close to the exceptional point.

The central new finding of Wang et al.^8^ is that the expected boost in sensitivity of the gyroscope is limited by a broadening of the two laser lines. The origin is extra quantum noise exceeding the quantum minimum by the so-called Petermann factor due to the nonorthogonality of the cavity modes^10^. Near the exceptional point the nonorthogonality is extreme (Fig. 1a) and the Petermann factor is therefore anticipated to be large. The experiment and the theoretical analysis of Wang et al. has shown that the large Petermann factor and the large signal-enhancement factor (essentially the splitting enhancement squared) precisely compensate each other. Consequently, the signal-to-noise ratio is not exceptional but rather conventional in this system.

## Prospects and future directions

The disappointing conclusion is that exceptional point-based sensing is per se not a way to circumvent the quantum-limited intensity noise. This is not completely unexpected, having in mind that sensing with exceptional points was originally introduced for classical wave systems^3^. However, there is no reason to be pessimistic. The theoretical studies^6,7^ have identified situations where exceptional point-based sensors can outperform conventional sensors also in the deep quantum domain. Moreover, the limits given by quantum intensity noise are less fundamental than those resulting from the uncertainty principle. The former can be beaten in quantum-enhanced measurements^11^ which use e.g. quantum entanglement as a resource. I believe that a combination of quantum-enhanced measurements and exceptional point-based sensing may open up an interesting new research direction.

The work of Wang et al.^8^ has also implications for the long-standing question about the laser linewidth exactly at the exceptional point. Modern laser theories that include the diverse linewidth corrections such as the Petermann factor and the Henry *α*-factor^12^ fail to give a definite answer here. From Petermann’s work one concludes that the laser linewidth is infinite directly at the exceptional point, but this appears to be unphysical. It was therefore a feeling of relief when a few years ago an experiment on phonon lasing observed a considerably broadened but finite laser linewidth at the exceptional point^13^. However, in contrast to the situation in a photon laser, the lasing phonon mode did not directly participate at the exceptional point but inherited the extra quantum noise from the degenerate photon modes. The experiment of Wang et al. now points back to the possibility of a diverging linewidth, even though an infinitely broadened linewidth has not been and cannot be directly measured. One reason is that the modes cannot be prepared exactly at the exceptional point. Thus it can always be claimed that when one goes closer to the exceptional point the linewidth broadening finally saturates. Future research has to clarify if such a regime exists and if enhanced sensitivity is possible in that regime.

Despite the observed limits, we will see further exciting and useful applications of exceptional point-based sensors, such as the already existing examples of sensor telemetry^14^ and implantable microsensors^15^. For such practical purposes, technical (classical) noise is more relevant than fundamental (quantum) noise. The influence of classical noise and how its impact can be minimized is not well enough understood yet. In this regard, a recent study has pointed to an insightful connection between exceptional points of Hamiltonians and that of Liouvillians governing the time evolution of density operators^16^. The Liouvillian exceptional points appear not exactly for the same parameter values due to the presence of quantum jumps^17^. Even if the quantum jumps can be ignored, the exceptional points are not the same, as the Liouvillian one is of higher order^16,17^, i.e. more Liouvillian eigenstates coalesce than energy eigenstates. While it is now known that the different order can lead to a noise-induced dynamical instability of exceptional point-based sensors^16^, the full implications of these differences are not identified yet. I assume that the exploration of this aspect, in particular in the context of a full quantum description of exceptional points, will become a focus in the research of non-Hermitian systems.

A huge potential for exceptional point-based sensors and non-Hermitian photonics lies in plasmonics^18^. The possibility to locally enhance the intensity of electromagnetic fields allows e.g. for nanoscale sensing. However, this application is challenged by unavoidable losses in plasmonic systems; non-Hermitian approaches may turn this disadvantage into an advantage.

A further promising avenue is the application of exceptional surfaces^19^. The strong response at an exceptional point requires low fabrication tolerances. Alternatively, a surface of exceptional points can be embedded in a higher-dimensional parameter space. Tailoring the system’s response such that the majority of fabrication errors and experimental uncertainties shift the operating point along the exceptional surface would make the system robust. At the same time enhanced energy splittings are possible if the target perturbation pushes the system away from the surface. It would be exciting to see this concept be realized experimentally.

Despite the enormous amount of theoretical and experimental work on exceptional points and non-Hermitian physics during the last decade, it seems that many aspects still remain unexplored. I expect that this rapidly advancing field will discover more counterintuitive consequences of non-Hermiticity and useful applications thereof in the near future.
